# Effects of Biotin-Avidin Interactions on Hydrogel Swelling

**DOI:** 10.3389/fchem.2020.593422

**Published:** 2020-11-19

**Authors:** Talaial B. Alina, Victoria A. Nash, Kara L. Spiller

**Affiliations:** ^1^Department of Chemical and Biological Engineering, Drexel University, Philadelphia, PA, United States; ^2^Biomaterials and Regenerative Medicine Laboratory, School of Biomedical Engineering, Science, and Health Systems, Drexel University, Philadelphia, PA, United States

**Keywords:** biotin-avidin, hydrogel, crosslinking, gelatin, swelling

## Abstract

Affinity interactions between the small molecule biotin and the protein avidin have been used extensively to functionalize biomaterials. More recently, researchers have leveraged the changes in biotin-avidin affinity that occur upon biotin conjugation to larger molecules to control the release of biotinylated drugs and proteins. However, the effects of biotin-avidin interactions on hydrogel properties have not been thoroughly investigated. The objective of this study was to evaluate the effect of increasing biotin and avidin concentrations on hydrogel swelling properties, as an indicator of crosslinking. Gelatin, selected as a model hydrogel material, was biotinylated at increasing fold molar excesses of biotin with a PEG linker using N-hydroxysuccinimide chemistry. Afterwards, biotinylated gelatin was formed into hydrogels and stabilized with glutaraldehyde. Swelling properties of the biotinylated hydrogels were investigated by conducting swelling studies in different avidin solutions. Increasing the degree of biotinylation caused significant decreases in swelling ratios of the hydrogels in a dose-dependent manner, suggesting increases in crosslinking of the hydrogels. However, increasing avidin concentrations in excess of biotin content did not significantly affect swelling ratios. Moving hydrogels to phosphate-buffered saline following avidin incorporation resulted in increased swelling ratios for hydrogels prepared with a lower concentration of biotin. However, hydrogels prepared with the highest concentration of biotin did not experience increased swelling ratios, implying that the stability of biotin-avidin-mediated crosslinking depends on the number of biotin molecules available for binding. Collectively, these results demonstrate that biotin-avidin interactions control hydrogel swelling properties, and that the magnitude and stability of the effects depend on the biotin concentration. These results have important implications for affinity-based controlled release of biotinylated drugs or proteins from biotin-avidin-crosslinked hydrogels.

## Introduction

Hydrogels are cross-linked networks of hydrophilic polymers and are extensively used in biomedical applications, such as in drug delivery and tissue engineering, due to their biophysical properties sharing similarities with tissues, relative biocompatibility, and their tunable degradability, strength, and porosity. This tunability arises from the multitude of ways that hydrogels can be crosslinked. Crosslinking determines mechanical properties, physiological stability, and diffusivity, an important parameter for drug and protein release. However, the level of control over drug or protein release achieved via diffusion is limited, with high burst release and relatively short durations of release.

As a result, affinity interactions between hydrogels and incorporated proteins have been explored to provide additional control. Affinity-controlled release of a protein involves reversible and non-covalent interactions between a protein and a binding ligand (Kastritis and Bonvin, 2013). For example, the glycosaminoglycan heparin has been conjugated onto different scaffolds and hydrogels to slowly release heparin-binding growth factors such as basic fibroblast growth factor (Pike et al., 2006; Yoon et al., 2006), nerve growth factor (Sakiyama-Elbert and Hubbell, 2000), and vascular endothelial growth factor (Pike et al., 2006; Tae et al., 2006) (for reviews, see Sakiyama-Elbert, 2014; Pakulska et al., 2016). In another study, Zhao et al. (2010) functionalized mineralized collagen bone matrix with antibodies having high specific binding for bone morphogenetic protein-2 (BMP2), demonstrating controlled BMP2 release (Zhao et al., 2010). In these systems, both binding affinity interactions and diffusion control protein release. However, most affinity binding systems are limited because they are only useful for certain affinity binding pairs and cannot be widely applied to all drugs or proteins (Tosh and Marangoni, 2004).

An exciting alternative to traditional affinity-based systems is the biotin and avidin binding pair, which bind with extremely high specificity and strength, because biotin is a small molecule that can be conjugated to virtually any polymer, drug, protein, or even cells with minimal effects on their bioactivity. Avidin is a protein derived from egg white that has a 67,000 MW and four binding sites for biotin (Green, 1975). With an extremely low dissociation constant (*K*_d_) of 10^−15^ M (Green, 1963a) and stability under different solvents, pH (Green, 1963b), and temperatures (Pritchard et al., 1966), biotin-avidin interactions have been used in a wide variety of applications (for review, see Jain and Cheng, 2017). However, conjugation of biotin to relatively large molecules, such as proteins or polymers, drastically reduces its binding affinity for avidin, suggesting the potential for affinity-based controlled release (Hofmann et al., 1982; Finn and Hofmann, 1985; Marek et al., 1997; Xiong et al., 2007; Spiller et al., 2015). This decrease in binding affinity could be attributed to steric hindrance impeding the ability of bound biotin to reach its binding sites on avidin (Ke et al., 2007). While a few studies have leveraged this reduced binding affinity for controlled release (Xiong et al., 2007; Spiller et al., 2015), the potential of this system remains underexplored. When compared to other affinity interactions, a major advantage of leveraging biotin-avidin interactions is that virtually any protein or polymer can be biotinylated, or conjugated to biotin, using a wide array of commercially available reagents without compromising the bioactivity of the protein (Wojda and Miller, 2000; Qureshi and Wong, 2002; Metzger et al., 2015). Thus, the release of biotinylated molecules from hydrogels theoretically can be controlled by affinity interactions as opposed to solely relying on diffusion. However, biotin-avidin interactions may affect hydrogel crosslinking, which would affect release, but these interactions have not been widely investigated.

Previous studies have demonstrated proof of concept that biotin-avidin interactions can cause crosslinking of hydrogels (Liu et al., 2010; Thompson et al., 2015). For example, Thompson et al. (2015) conjugated biotinylated peptides to poly(ethylene glycol) (PEG) chains using Michael addition. These biotin-peptide-PEG conjugates were then mixed with avidin to form crosslinked hydrogels. The mechanical properties of the hydrogels, and therefore their crosslinking densities, were dependent on the concentration of biotinylated polymer (Thompson et al., 2015). In another study, Cui et al. (2013) biotinylated hyaluronic acid hydrogels and loaded them with avidin and the chemotherapeutic drug doxorubicin. Excess biotin was then used to disrupt interactions between biotinylated polymer and avidin in order to release loaded doxorubicin from this system via diffusion (Cui et al., 2013). While these studies highlighted how biotin-avidin can be used to crosslink a hydrogel to affect drug release, the effects of biotin concentration on hydrogel swelling or the stability of the biotin-avidin interactions were not explored.

**Figure 1:**
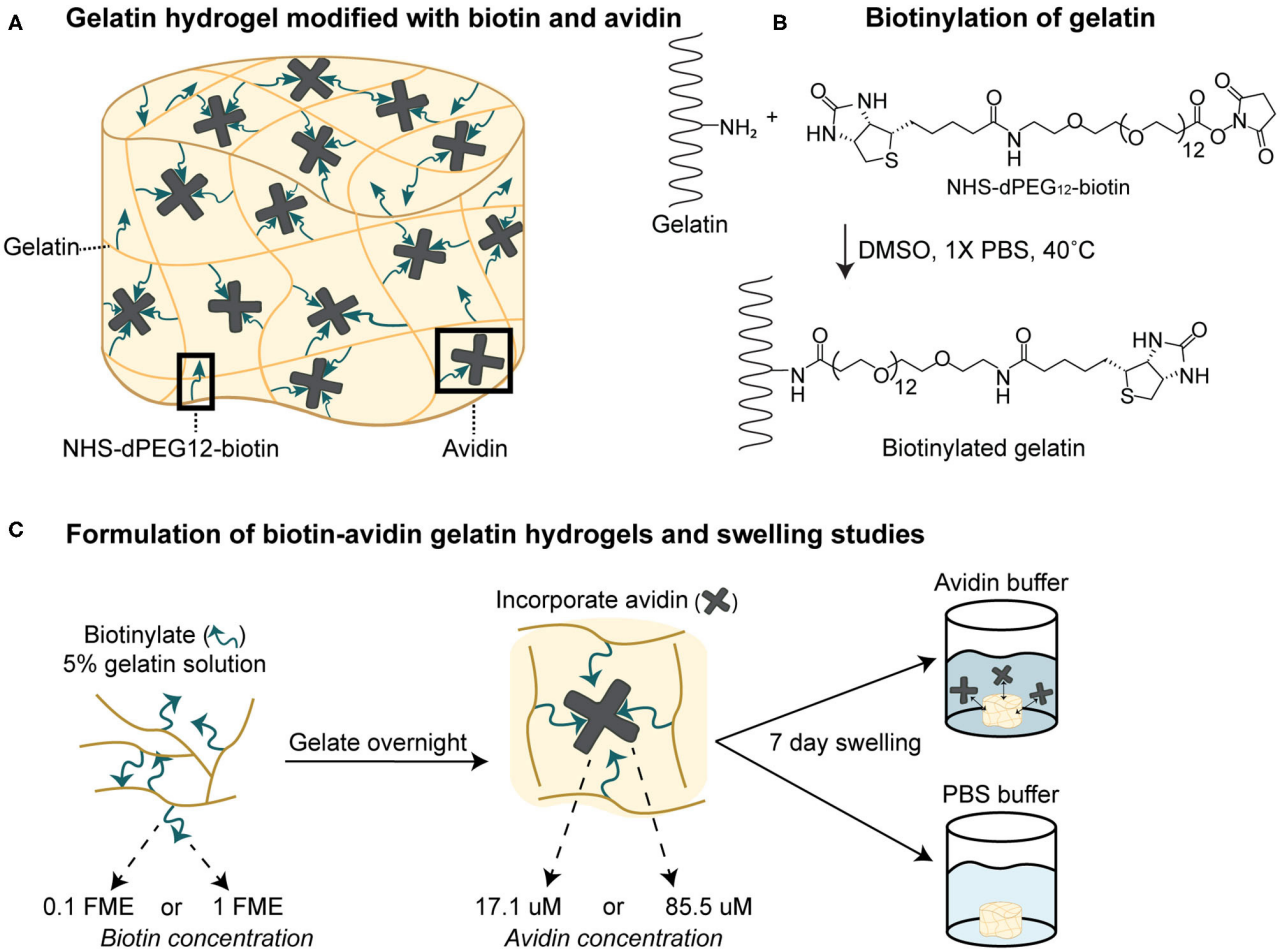
Overview of the experimental design. **(A)** Gelatin hydrogels were modified with NHS-dPEG_12_-biotin and loaded with avidin to investigate biotin-avidin-mediated crosslinking. **(B)** Gelatin polymers were biotinylated by reaction with NHS-dPEG_12_-biotin via conjugation to primary amines on gelatin. MarvinSketch was used for illustrating chemical structures, substructures and reactions, Marvin 20.15.0, 2020, ChemAxon (http://www.chemaxon.com). **(C)** Hydrogels were formed by allowing biotinylated gelatin polymers, prepared with 0.1 or 1 fold molar excesses (FME) of biotinylation reagent, to gelate overnight. Then, they were either placed directly into 17.1 or 85.5 μM avidin solutions for swelling studies in the presence of avidin or incubated overnight in 85.5 μM avidin prior to swelling studies in PBS to evaluate biotin-avidin stability.

Overall, previous studies have provided evidence that biotin-avidin interactions can affect hydrogel crosslinking, with implications for controlled release systems. However, there are few reports on how changing biotin-avidin parameters affect hydrogel crosslinking or swelling, which is inversely related to crosslinking. Therefore, there exists a need to systematically study biotin-avidin swelling and controlled release. This study investigated those effects by testing how increasing biotin concentration affects hydrogel swelling and whether biotin-avidin interactions persist over time (Figure 1). Gelatin hydrogels (Figure 1A) were prepared and biotinylated with varying fold molar excess (FME) of NHS-dPEG_12_-biotin (Figure 1B). Swelling studies were performed in avidin to determine the effects of time-dependent biotin-avidin interactions, as well as in phosphate-buffered saline (PBS) to determine if biotin-avidin binding dissociates over time (Figure 1C).

## Materials and Methods

### Modeling Effect of FME on Biotin Binding Efficiency for Gelatin Hydrogels

To understand how FME could be used to modulate the amount of NHS-dPEG_12_-biotin (Quanta BioDesign) conjugated to gelatin hydrogels, a model molecule of NHS-DyLight 350 (Thermo Fisher Scientific) was used. First, 1 mg/mL aliquots of NHS-DyLight 350 prepared in dimethyl sulfoxide (DMSO) were equilibrated to room temperature. Then, these aliquots were diluted in 1X PBS (Fisher Scientific) to create 0, 0.01, 0.1, and 1 FME Dylight 350 solutions. Next, Dylight 350 was proportionally added based on the number of primary amines on gelatin, assuming 1.64 × 10^−4^ mmol per 50,000 MW gelatin polymer. This assumption was based on an average of values reported in literature (Bubnis and Ofner, 1992). Then, dry gelatin derived from bovine skin (225 g Bloom, Type B, Sigma Aldrich) was added to these solutions to create 5% gelatin-DyLight 350 solutions in individual beakers. These beakers were stirred at 500 rpm at 40°C for an hour. Next, these solutions were added to dialysis cassettes (Thermo Fisher Scientific 3.5K MWCO) and dialyzed for 2 h in 1X PBS at room temperature. After 2 h, the dialysis buffer was removed and replaced with fresh buffer. The solutions were then dialyzed for an additional 18 h. Then, biotinylated gelatin solutions were cast in wells of a 96-well cell culture plate (Corning Costar 96-well Cell Culture Plate). To measure the resulting fluorescence, casted gelatin-DyLight 350 solutions were read on a BioTek plate reader using an excitation wavelength of 353 nm and read at an emission wavelength of 432 nm. The concentration of DyLight 350 conjugated to gelatin was interpolated from a standard curve, which was prepared from a serial dilution of 25 μg/mL DyLight 350. Binding efficiency was calculated by comparing the values of bound DyLight 350 to the amount added to the gelatin-DyLight 350 solutions before dialyzing them. Assuming a similar binding efficiency of NHS-dPEG_12_-biotin, the maximum theoretical numbers of available binding sites for avidin was calculated for 1–4 mol biotin bound to each mol avidin.

### Preparation of Biotinylated Gelatin Hydrogels

Gelatin derived from bovine skin was used to prepare hydrogel samples based on modified methods (Ratnikov et al., 2000; Bigi et al., 2001; Yu et al., 2016). After estimating the number of amine groups present as described above, a 25 mM stock solution of NHS-dPEG_12_-biotin was prepared in DMSO (Sigma-Aldrich). Then, 0, 0.1, and 1 FME biotin solutions were created in different tubes by diluting the stock biotin solution in 1X PBS. Next, dry gelatin was added to each biotin solution to form 5% gelatin solutions. These solutions were vortexed at low speeds and then incubated for 1 h in a water bath at 40°C, which is above the gelation temperature of gelatin but below the denaturation point of biotin.

After an hour, biotinylated gelatin solutions were revortexed at low speeds, added to dialysis cassettes (Thermo Fisher Scientific 3.5K MWCO Dialysis Cassettes), and dialyzed for 2 h in 1X PBS at room temperature. After 2 h, the dialysis buffer was removed and replaced with fresh buffer. The solutions were then dialyzed for an additional 18 h. After 18 h, biotinylated gelatin solutions were cast in wells of a 24-well cell culture plate (Corning Costar 24-well Cell Culture Plate). After 24 h of gelation at 4°C, 5 mm biopsy punches (Integra Miltex) were used to extract individual hydrogel disc from the wells. Biotinylated gelatin hydrogels were then stabilized by incubation in 200 μL of prepared 0.05% glutaraldehyde (Sigma-Aldrich) overnight in individual tubes, shaking at 100 RPM and 25°C. Next, excess glutaraldehyde was quenched by incubating the hydrogels in 200 μL of prepared 0.1 M glycine (pH 7.4, Thomas Scientific) overnight, shaking at 100 RPM and 25°C. Afterwards, the samples were soaked in 200 μL of 1X PBS overnight, shaking at 100 RPM and 25°C, to remove excess glycine.

### Dry Mass Measurements of Biotinylated Hydrogels

After preparing the hydrogels, representative hydrogels from 0, 0.1, and 1 FME groups were placed in individual tubes, frozen overnight, and then lyophilized (Labconco Freezone Freeze Dry System) overnight. Next, lyophilized hydrogels were individually massed (Sartorius Quintix 64-1S Analytical Balance) to obtain dry mass data (*W*_*d*_).

### Swelling Studies

To study the effect of biotin concentration on swelling properties of biotinylated hydrogels, biotinylated hydrogels were placed in 1 mL of 17.1 μM (1.15 mg) or 85.5 μM (5.73 mg) avidin (Thermo Fisher Scientific) swelling buffer for 7 days. Every 24 h, biotinylated hydrogels were removed from the swelling solution, blotted to remove excess liquid, and placed in an Eppendorf tube to measure their swollen mass (*W*_*s*_). Their masses were compared to initial dry mass (*W*_*d*_) to calculate their swelling ratios (*Q*_*m*_) using Equation (1). Swelling ratio data were normalized to the day 0 time point to represent fold change over time and then log transformed.

(1) $$\begin{array}{l} Q_{m}=\frac{W_{s}-W_{d}}{W_{d}}\times 100\% \end{array}$$

To study the stability of biotin-avidin binding, biotinylated gelatin hydrogels were first swollen overnight in 200 μL of 85.5 μM avidin and then soaked in 200 μL of 1X PBS to remove excess avidin before being transferred to PBS for 7 days.

### Statistical Analysis

Statistical analysis was conducted in GraphPad 8.4.3. Data were first tested for normality using a QQ plot. One-way ANOVA with Tukey's *post-hoc* test was used to compare DyLight binding based on FME biotinylation. Two-way ANOVA with the Geisser-Greenhouse correction and either Sidak's or Tukey's *post-hoc* test was used, as appropriate. Sidak's *post-hoc* test was used to compare the swelling ratios of three different FME hydrogels in 17.1 or 85.5 μM avidin for 7 days, while Tukey's *post-hoc* test was used to compare the effects of time on swelling ratios of three different FME hydrogels in avidin or in PBS.

## Results

### Characterization of Biotinylated Hydrogels

**Figure 2:**
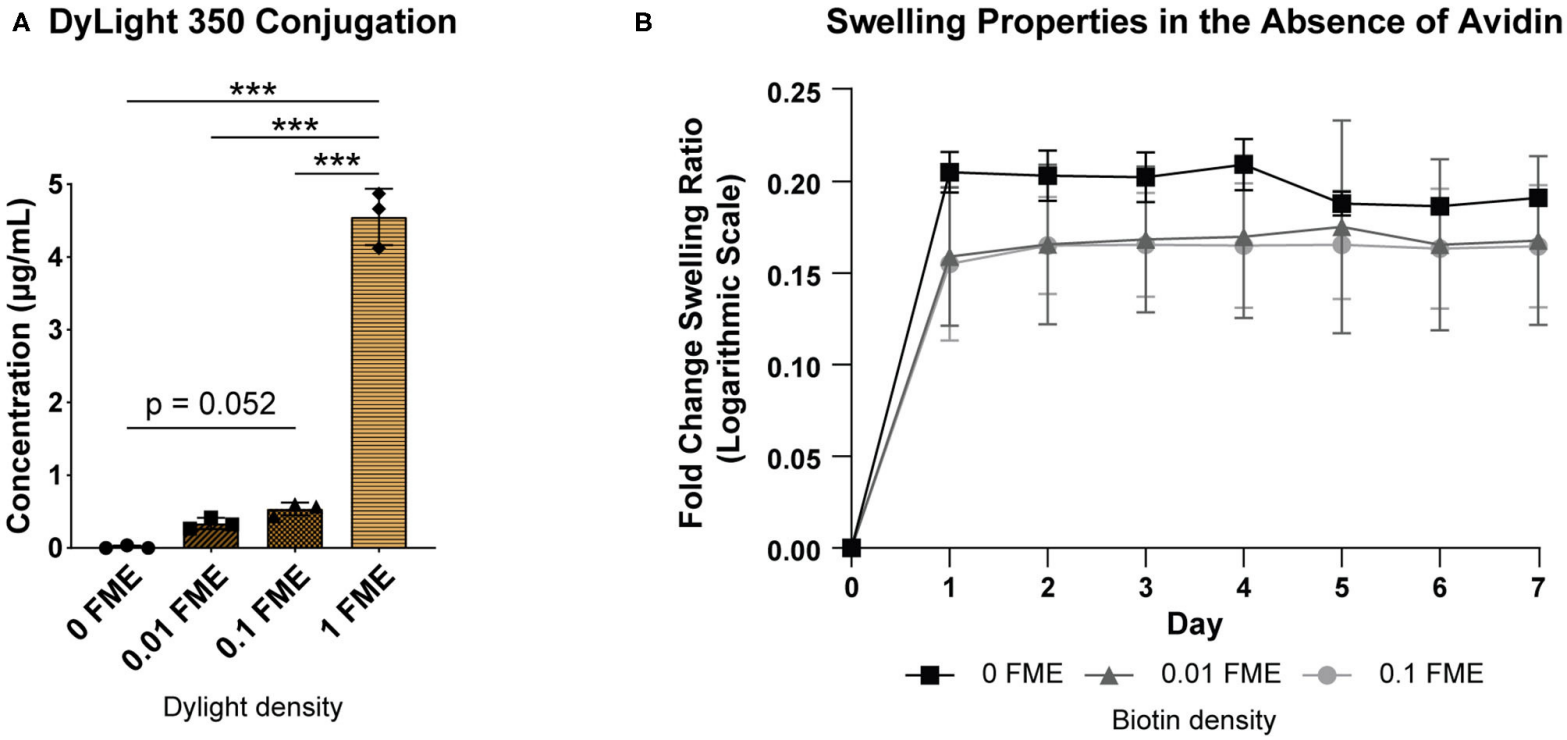
Characterization of the effects of biotinylation on hydrogel properties. **(A)** Concentration of bound DyLight 350 in hydrogels prepared with 0.01, 0.1, and 1 FME NHS-DyLight 350. *n* = 3, mean ± SD. One-way ANOVA with Tukey's *post-hoc* test (***p < 0.001). (**B**) Swelling ratios of biotinylated hydrogels in PBS over 7 days. *n* = 2, mean ± SD. No statistical significance between groups or time points, determined using a two-way ANOVA with multiple comparisons *post-hoc* Tukey test.

With the same binding moiety as NHS-dPEG_12_-biotin, NHS-DyLight 350 was chosen to model how increasing the concentration of binding reagent would affect binding efficiency to gelatin. Increasing the FME of DyLight 350 in a 5% gelatin solution resulted in increasing amounts of the molecule bound to gelatin (Figure 2A). The swelling ratios of 0, 0.01, and 0.1 FME biotinylated hydrogels in PBS, without avidin, were measured to determine if biotin concentration affected their swelling properties. The swelling ratios of non-biotinylated hydrogels significantly increased between day 0 and 4 and then stayed constant for 3 more days whereas the swelling ratios of hydrogels biotinylated with 0.01- or 0.1-FME biotin did not significantly change between days (Figure 2B, Supplementary Figure 1). There were no significant differences between groups at any time point.

### Effects of Biotin Concentration on Swelling Properties in the Presence of Avidin

**Figure 3:**
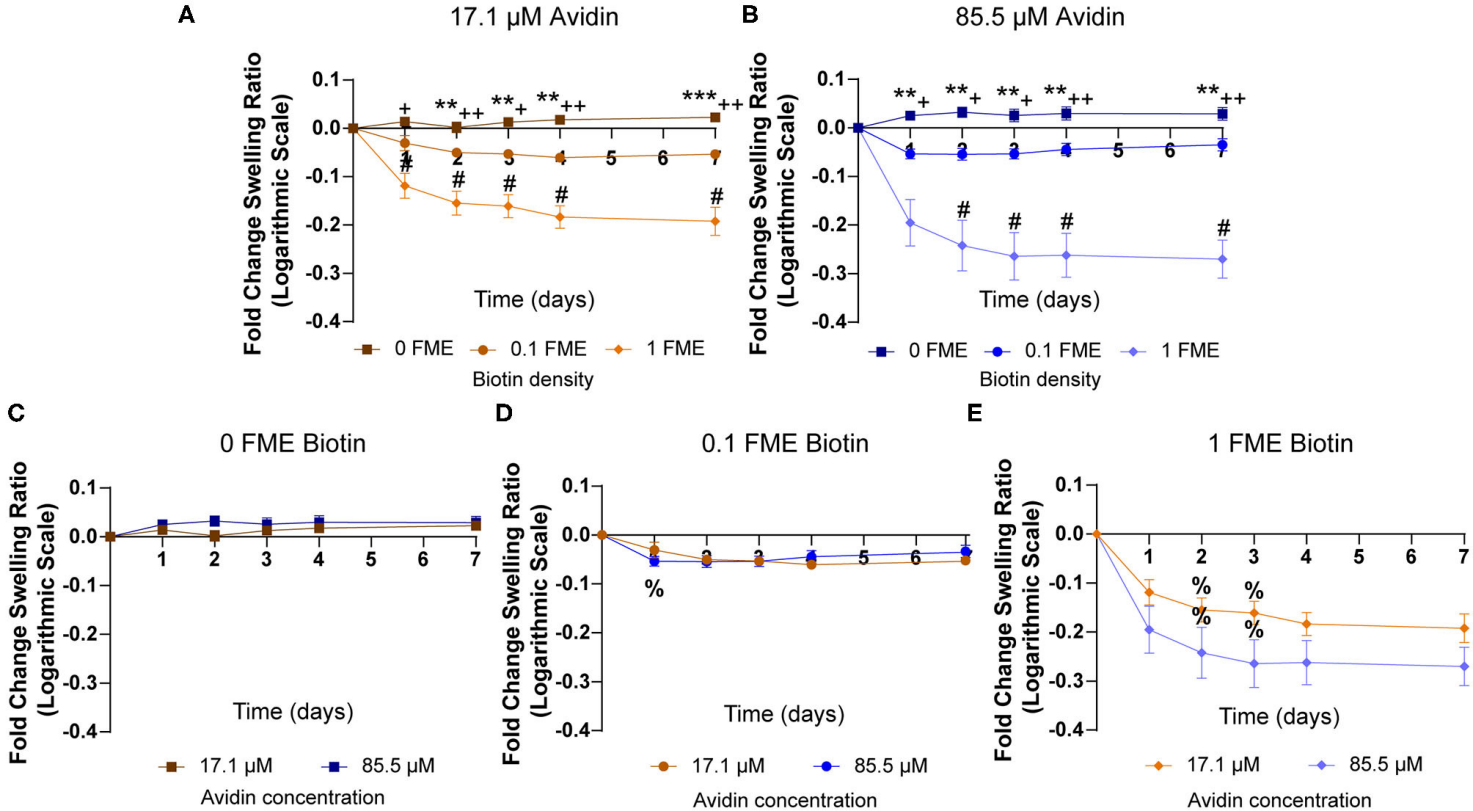
Effects of biotin concentration on swelling properties in avidin swelling buffer. **(A,B)** Fold changes in swelling ratios compared to day 0 time point of hydrogels biotinylated with 0, 0.1, or 1 FME NHS-dPEG_12_-biotin in **(A)** 17.1 μM avidin or **(B)** 85.5 μM avidin for 7 days. *n* = 3, mean ± SD. Statistical significance between different FME hydrogels determined using two-way ANOVA with Tukey's *post-hoc* test (0 FME vs. 0.1 FME: **p < 0.01, ***p < 0.001; 0 FME vs. 1 FME: ^+^*p* < 0.05, ^++^*p* < 0.01; 0.1 FME vs. 1 FME: ^#^*p* < 0.05). **(C–E)** Fold changes in swelling ratios of hydrogels with **(C)** 0 FME, **(D)** 0.1 FME, or **(E)** 1 FME NHS-dPEG_12_-biotin, in either 17.1 or 85.5 μM avidin solutions, over 7 days. *n* = 3, mean ± SD. No statistical significance resulting from different avidin concentrations, determined using two-way ANOVA with Sidak's *post-hoc* test. Statistical significance between time points determined using two-way ANOVA with Tukey's *post-hoc* test (^%^indicates *p* < 0.05 compared to the previous day).

To investigate the effect of biotin-avidin binding on hydrogel swelling, as an indirect measurement related to crosslinking, biotinylated gelatin hydrogels were swollen in avidin solutions for 7 days. Hydrogels prepared with 0.1 or 1 FME biotin decreased in swelling ratio within 1 day, with significantly lower swelling ratios compared to non-biotinylated (0 FME) hydrogels (Figure 3A, Supplementary Figure 2A). These trends were more pronounced when the hydrogels were incubated in 85.5 μM avidin swelling buffer (Figure 3B, Supplementary Figure 2B), although there were no significant differences in swelling ratio as a result of avidin concentration (Figures 3C–E, Supplementary Figures 2C–E). However, it is important to note that both avidin concentrations were well above the theoretical binding capacity of biotin bound to the hydrogels (Table 1).

**Table 1:** Theoretical avidin binding in biotinylated gelatin hydrogels, assuming 1:1, 1:2, 1:3, and 1:4 avidin to biotin binding.

**Mol avidin : mol NHS-dPEG_**12**_-biotin**	**1:1**	**1:2**	**1:3**	**1:4**
**FME biotin**	**Theoretical avidin binding per hydrogel (pmol)**			
0.1	14.1 ± 1.5	7.07 ± 0.74	4.71 ± 0.50	3.53 ± 0.37
1	198 ± 30	99.0 ± 14.9	66.0 ± 9.9	49.5 ± 7.5
Avidin supplied:				
17,100 pmol (17.1 μM, 1.15 mg)/85,500 pmol (85.5 μM, 5.73 mg)				

### Stability of Biotin-Avidin Interactions When Swelling in PBS

**Figure 4:**
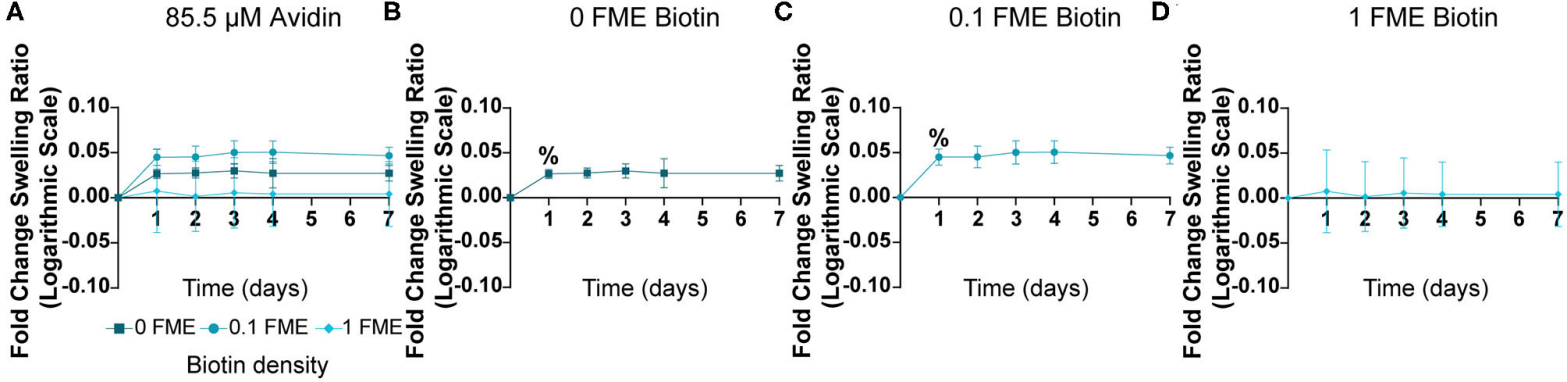
Stability of biotin-avidin hydrogels in PBS following overnight incubation in 85.5 μM avidin solution. **(A–D)** Fold changes in swelling ratios of hydrogels, with figures in **(B–D)** illustrating changes over time. *n* = 3, mean ± SD. No statistical significance resulting from FME, determined using two-way ANOVA with Tukey's *post-hoc* test. Statistical significance between time points determined using two-way ANOVA with Tukey's *post-hoc* test (^%^indicates *p* < 0.05 compared to the initial time point).

Having confirmed that biotin-avidin interactions affect hydrogel swelling properties, we next sought to determine the stability of this interaction by transferring the hydrogels to PBS following overnight incubation in avidin solution. Hydrogels prepared with no biotin or with 0.1 FME biotinylation significantly increased in swelling ratio within 24 h, although there were no significant differences between these two groups (Figures 4A–C, Supplementary Figures 3A–C). In contrast, hydrogels biotinylated with 1-FME showed no change in swelling ratio upon incubation in PBS (Figures 4A,D, Supplementary Figures 3A,D).

## Discussion

This study shows that biotin-avidin interactions affect hydrogel swelling properties, suggesting their potential as a novel technique to control hydrogel crosslinking. While previous studies have demonstrated that biotin-avidin interactions can cause hydrogel crosslinking, the effects of biotin concentration on hydrogel swelling or its potential to bind and release avidin have not been explored. Here, we found that increasing biotin concentration resulted in decreased swelling, suggesting increased crosslinking, as well as increased stability of the biotin-avidin interaction. These findings show that biotin-avidin interactions can affect the functionality of a hydrogel as a biomaterial, with implications for controlled release of biotinylated and non-biotinylated drugs.

While numerous affinity binding systems have been incorporated into hydrogels to control the release of drug or proteins, none have the modularity, versatility, and high specificity and strength of the biotin-avidin system. However, they also would not be expected to affect hydrogel crosslinking since they typically employ just one binding interaction. In contrast, biotin can bind to four binding sites on avidin, resulting in the potential for hydrogel crosslinking if one avidin binds multiple biotin molecules bound to nearby polymer chains. Indeed, we found that increasing biotin concentration led to decreased swelling ratio, suggesting increased probability of a given avidin molecule binding to at least two biotin molecules on adjacent chains. These results are important since hydrogel swelling and crosslinking affect gel formation, mechanical properties, degradability, and diffusivity of molecules like drugs or nutrients, as in the case of hydrogels used in tissue engineering. Hydrogel swelling properties are also important for maintaining stability when implanted into tissue environments characterized by high osmotic pressures (Spiller et al., 2009).

To our knowledge, the first report that biotin-avidin interactions could cause hydrogel crosslinking was described by Liu et al. (2010) in which biotinylated PEG oligomers were mixed with avidin. Their reaction resulted in their transition from solution to gel (Liu et al., 2010). Thompson et al. (2015) later showed that this crosslinking effect was dependent on the concentration of biotinylated polymer, which also affected the stability of the hydrogels, in that hydrogels prepared with higher biotinylated polymer concentration eroded more slowly in PBS than hydrogels prepared at lower concentrations (Thompson et al., 2015). Those results suggested dissociation of the biotin-avidin interaction, which is a well-described consequence of the reduced affinity that occurs upon conjugation of biotin to relatively large molecules like proteins or polymers (Hofmann et al., 1982; Finn and Hofmann, 1985; Kaiser et al., 1997; Ke et al., 2007; Xiong et al., 2007; Spiller et al., 2015). Interestingly, we found the stability of the biotin-avidin interactions to be dependent on biotin concentration because hydrogels prepared with the highest biotin concentration and 1 day of incubation with avidin did not significantly change in swelling ratio over 7 days in PBS. In contrast, hydrogels prepared with the lower biotin concentration or without biotin both showed increases in swelling ratio within 1 day of being transferred to PBS, suggesting decreased crosslinking, presumably because of avidin diffusing from the hydrogels. The fact that even hydrogels prepared without biotin showed a decrease in swelling ratio suggests that avidin may have formed nonspecific crosslinks with surrounding polymer chains. Alternatively, the diffusion of avidin from these hydrogels may have resulted in a decrease in osmotic pressure, which would cause swelling. It was interesting that biotin-avidin interactions were more stable in the hydrogels with more biotin (1 FME) compared to less biotin (0.1 FME), even though both showed significant effects of biotin-avidin interactions on swelling properties. These results may suggest that each avidin molecule may have been bound to more than 2 biotin molecules in the higher biotin concentration hydrogels but not the lower, such that the dissociation of one bond did not result in a change in swelling because two other biotin molecules remained bound. Alternatively, avidin molecules may have encountered more biotin molecules upon diffusion out of the hydrogels, resulting in insignificant changes in swelling properties at the macroscale. Further studies of molecular binding interactions are required to investigate the mechanism behind this finding.

Beyond the effects of biotin-avidin interactions on the biophysical properties of hydrogels, these results have important implications for affinity-based controlled release of biotinylated drugs or proteins. To our knowledge, Xiong et al. (2007) was the first to use this affinity interaction system for delivery of bioactive molecules from biomaterials. They created a long circulating poly(ethylene glycol) (PEG) nanoparticle, decorated with the cationic polymer poly(ethyleneimine) (PEI) via biotin-avidin interactions for gene delivery applications. Controlled release of PEI could then be achieved by adding excess free biotin to the system, which displaced the biotinylated-PEI due to its lower affinity for avidin compared to free biotin. The potential to control release of biotinylated drugs from biotinylated scaffolds was later introduced by Spiller et al. (2015), although detailed investigations into the effects of biotin-avidin interactions on drug delivery were not conducted. In the present study, biotin-avidin interactions were found to affect hydrogel swelling, indicating that at least some biotin-binding sites were not available for binding to biotinylated drugs or proteins. Especially if the binding affinity between avidin and biotinylated hydrogel was substantially higher than the binding affinity between avidin and biotinylated drug, then the release of biotinylated drug would depend solely on diffusion and not biotin-avidin interactions. Thus, the release of biotinylated drug or protein from biotinylated hydrogels containing avidin would be expected to depend on (1) the relative concentrations of biotin and avidin, (2) the relative differences in binding affinity between avidin-biotinylated polymer and avidin-biotinylated drug, and (3) even the order of hydrogel fabrication, in terms of whether avidin is allowed to first associate with biotinylated drug prior to incorporation into the hydrogel or whether biotinylated drug is added to a biotinylated hydrogel that is first crosslinked with avidin.

In summary, biotin-avidin interactions potentially allow for a wide range of control over the rates of release of biotinylated drugs or proteins from biotinylated hydrogels. Future work should focus on more direct investigations of hydrogel crosslinking, in addition to macroscopic observations of hydrogel swelling, as well as the swelling mechanisms.

## Conclusions

Biotin-avidin interactions were shown to affect hydrogel properties through swelling studies. Increasing biotin concentration resulted in decreased swelling ratios, suggesting increased crosslinking. Additionally, the stability of the biotin-avidin interactions depended on biotin concentration, with hydrogels prepared with the highest biotin concentration showing no changes in swelling ratio upon transferring to PBS, unlike hydrogels with lower biotin densities. Overall, this study demonstrates that biotin-avidin binding can affect hydrogel swelling properties, with implications for biomaterial functionality and protein release.

## Data Availability Statement

The raw data supporting the conclusions of this article will be made available by the authors, without undue reservation.

## Author Contributions

TA conducted and analyzed the experiments and wrote the manuscript. VN assisted in experimental design, interpretation of results, and figure and manuscript preparation. KS conceived and supervised the research and assisted in manuscript preparation. All authors contributed to the article and approved the submitted version.

## Conflict of Interest

The authors declare that the research was conducted in the absence of any commercial or financial relationships that could be construed as a potential conflict of interest.
