# Supplementary material for: Effects of Biotin-Avidin Interactions on Hydrogel Swelling
Source: Front Chem. 2020 Nov 19;8:593422. doi: 10.3389/fchem.2020.593422 (PMC7711042; doi:10.3389/fchem.2020.593422)
Supplement: Supplementary file 1 [file Data_Sheet_1.PDF]

Supplementary Material

Effects of Biotin-Avidin Interactions on Hydrogel Crosslinking

Talaial B. Alina, Victoria A. Nash, Kara L. Spiller

1 Supplementary Figures

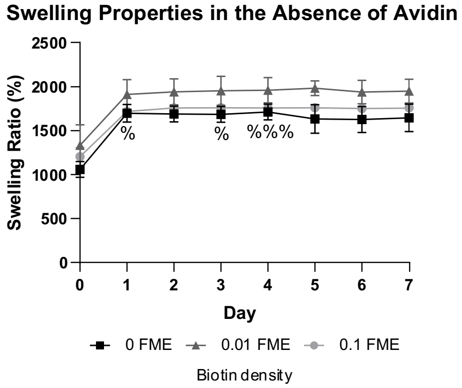

Supplementary Figure 1. Swelling ratios of biotinylated hydrogels in PBS over 7 days. n = 2, mean ± SD. No statistical significance between groups resulting from different FME, determined using two-way ANOVA with Tukey's post hoc test. Statistical significance between time points determined using two-way ANOVA with Tukey's post hoc test ([%]indicates $p < 0.05$ and [%%%]indicates $p < 0.001$ compared to the previous day for the 0 FME group).

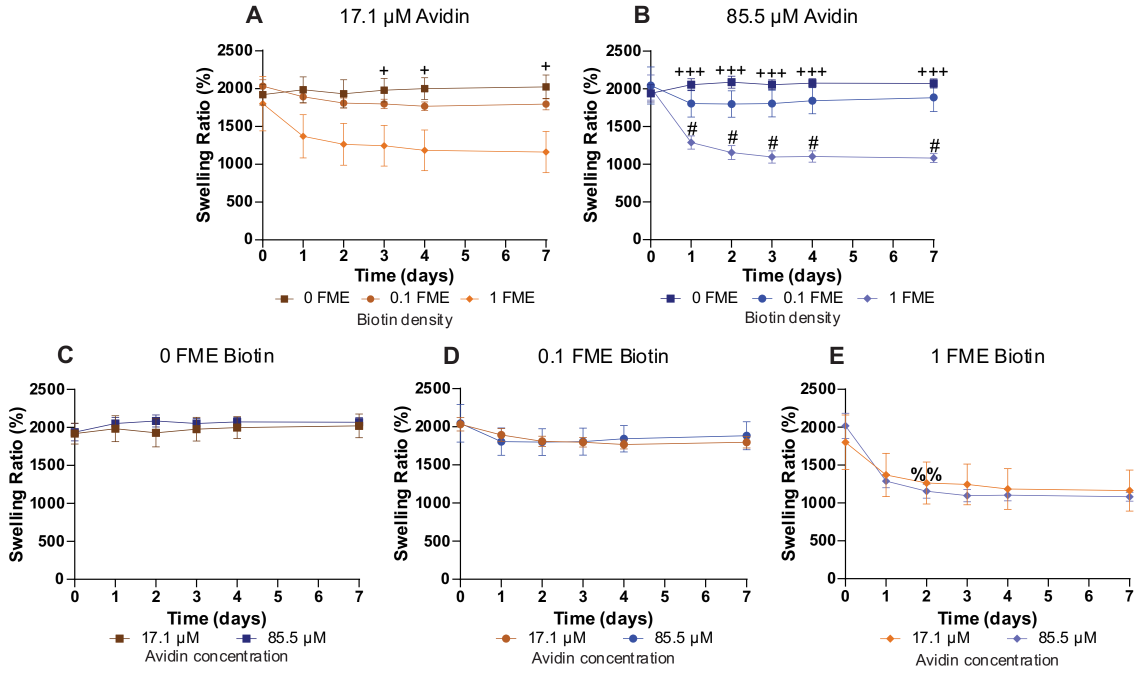

Supplementary Figure 2. Effects of biotin density on swelling properties in avidin swelling buffer. (**A-B**) Fold changes in swelling ratios compared to day 0 time point of hydrogels biotinylated with 0, 0.1, or 1 FME NHS-dPEG$_{12}$-biotin in (**A**) 17.1 μM avidin or (**B**) 85.5 μM avidin for 7 days. n = 3, mean ± SD. Statistical significance between different FME hydrogels determined using two-way ANOVA with Tukey's post hoc test (0 FME versus 1 FME: $^{+}p < 0.05$, $^{+++}p < 0.001$; 0.1 FME versus 1 FME: $^{\#}p < 0.05$). (**C-E**) Swelling ratios of hydrogels with (**C**) 0 FME, (**D**) 0.1 FME, or (**E**) 1 FME NHS-dPEG$_{12}$-biotin, in either 17.1 μM or 85.5 μM avidin solutions, over 7 days. n = 3, mean ± SD. No statistical significance resulting from different avidin concentrations, determined using two-way ANOVA with Sidak's post hoc test. Statistical significance between time points determined using two-way ANOVA with Tukey's post hoc test ($^{\%\%}$indicates $p < 0.01$ compared to the previous day for the 85.5 μM avidin group).

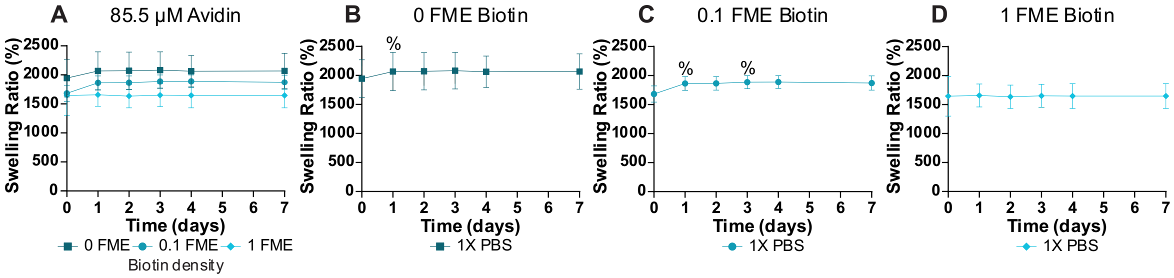

Supplementary Figure 3. Stability of biotin-avidin hydrogels in PBS following overnight incubation in 85.5 μM avidin solution. (**A-D**) Fold changes in swelling ratios of hydrogels, with figures in (**B-D**) illustrating changes over time. n = 3, mean ± SD. No statistical significance resulting from FME, determined using two-way ANOVA with Tukey's post hoc test. Statistical significance between time points determined using two-way ANOVA with Tukey's post hoc test ($^{\%}$indicates $p < 0.05$ compared to the previous day).
